# Analysis of six aromatic amines in the mainstream smoke of tobacco products

**DOI:** 10.1007/s00216-022-04075-7

**Published:** 2022-04-11

**Authors:** Huihua Ji, Zhenyu Jin

**Affiliations:** Kentucky Tobacco Research and Development Center, University of Kentucky, Lexington, KY 40546 USA

**Keywords:** Aromatic amines, Tobacco smoke, Solid-phase microextraction, Gas chromatography-triple quadrupole mass spectrometry

## Abstract

Aromatic amines are a class of carcinogenic compounds in tobacco smoke that are listed on the FDA list of harmful and potentially harmful constituents (HPHCs). A method using solid-phase microextraction-coupled to gas chromatography-triple quadrupole mass spectrometry (SPME headspace GC–MS/MS) was developed and validated for the quantitative determination of six aromatic amines, including 1-aminonaphthalene (1-AN), 2-aminonaphthalene (2-AN), 3-aminobiphenyl (3-ABP), 4-aminobiphenyl (4-ABP), o-toluidine (o-TOL), and o-anisidine (o-ANI), in the mainstream smoke of cigarettes, cigars, and heated tobacco products. The method developed here combines high sensitivity with simple sample preparation and has demonstrated satisfactory linearity for all six aromatic amines with correlation coefficients greater than 0.9994. The limits of detection range and the limits of quantitation range were 12–96 pg/mL and 41–320 pg/mL, respectively. Their recoveries and coefficients of variation (CV%) were 90–112% and 2.1–6.6%, respectively. The new SPME headspace GC/MS/MS method has been successfully applied to measure the contents of the six aromatic amines in the mainstream smoke of cigarettes, cigars, and heated tobacco products.

## Introduction

Tobacco smoking causes approximately 20% of total cancers and around 30% of total cancer deaths in the USA [1]. Tobacco smoke is a complex mixture that contains thousands of compounds, including more than 60 carcinogens that were identified by the year 2000 [2]. Some aromatic amines are included in this group of carcinogens. In March 2012, the FDA established a list of harmful and potentially harmful constituents (HPHCs) in tobacco products and tobacco smoke [3]. The aromatic amines, including 1-aminonaphthalene (1-AN), 2-aminonaphthalene (2-AN), and 4-aminobiphenyl (4-ABP), are on the FDA HPHCs list. The International Agency for Research on Cancer (IARC) has classified 2-AN, 4-ABP, and o-toluidine (o-TOL) as group 1 carcinogens, carcinogenic to humans, while o-anisidine (o-ANI) in group 2A is probably a human carcinogen [4]. Exposure to the aromatic amines, such as 4-ABP, 2-AN, and o-TOL, can cause urinary bladder cancer in humans and cause tumors at various sites in laboratory animals [2, 5, 6].

Hoffmann and Masuda used gas chromatography with an electron capture detector (GC-ECD) to determine 1-AN and 2-AN in mainstream cigarette smoke in 1969 [7]. Since that time, several methods for determining aromatic amines in mainstream or sidestream cigarette smoke have been published [8–13]. These methods involved various techniques such as gas chromatography-mass spectrometry (GC/MS) [7–10], gas chromatography-triple quadrupole mass spectrometry with negative ion chemical ionization mode (GC/MS/MS-NICI) [11, 12], and liquid chromatography-electrospray ionization tandem mass spectrometry (HPLC/MS/MS) [13]. Due to the low concentration of aromatic amines in cigarette smoke, these methods usually involved complicated sample preparation procedures, such as evaporation of the solvent to pre-concentrate the solution, and liquid-liquid extraction, among others.

Solid-phase microextraction (SPME) is a sampling and sample preparation technique in which the analytes are absorbed and desorbed onto the fiber stationary phase [14]. SPME combines analyte sampling, isolation, and enrichment into one simple step. It is a simple, quick, solvent-free, and inexpensive extraction technique. The SPME technique is widely used in a variety of fields, such as aroma studies, food and environmental analysis, forensic and pharmaceutical samples analysis, and bioanalytical applications [14, 15]. However, to the best of our knowledge, there are no published studies reporting the use of SPME to determine aromatic amines in tobacco smoke. In this study, the SPME headspace GC-MS/MS method was developed for the quantitative determination of aromatic amines, including 1-AN, 2-AN, 3-ABP, 4-ABP, o-TOL, and o-ANI, in tobacco smoke. The advantage of this method is high sensitivity with simple sample preparation. The method developed here has been successfully applied to determine the aromatic amines in the mainstream smoke of cigarettes, cigars, and heated tobacco products (HTPs).

## Experimental

### Reagents and materials

The chemical standards 1-AN (purity ≥ 99%), 3-aminobiphenyl (3-ABP, purity > 97%), 4-ABP (purity ≥ 98%), o-TOL (purity ≥ 99%), o‑ANI (purity ≥ 99%), and N-methyl-bis(trifluoroacetamide) (MBTFA) were purchased from MilliporeSigma (St. Louis, MO). 1 mg/mL 2-AN in benzene was purchased from Cambridge Isotope Laboratories, Inc. (Tewksbury, MA). Isotopically labeled 1-aminonaphthalene-d_7_ (1-AN-d_7,_ 99.3%-d_7_), 2-aminonaphthalene-d_7_ (2-AN-d_7,_ 98.4%-d_7_), 4-aminobiphenyl-d_9_ (4-ABP-d_9,_ 99.5%-d_9_), ortho-toluidine-d_9_ (o-TOL-d_9,_ 98.9%-d_9_), and o‑anisidine-d_7_ (o-ANI-d_7,_ 99.4%-d_7_), the internal standards, were purchased from CDN Isotopes Inc. (Quebec, Canada). 3-Aminobiphenyl-d_9_ (3-ABP-d_9_) was from Toronto Research Chemicals (Toronto, Canada). All other reagents were obtained from Fisher Scientific (Hampton, NH). The SPME fibers divinylbenzene/carboxen/polydimethylsiloxane (DVB/CAR/PDMS) 50/30 µm, polydimethylsiloxane/divinylbenzene (PDMS/DVB) 65 µm, carboxen/polydimethylsiloxane (CAR/PDMS) 85 µm, polyacrylate 85 µm, and polyethylene glycol (PEG) 60 µm were purchased from Supelco-MilliporeSigma (Bellefonte, PA).

### Samples

The certified reference cigarette 1R6F, the reference cigarette 2R5F, and the four reference cigars including the machine-made large cigar (1C1), the filtered cigar (1C2), the cigarillo (1C3), and the large cigar with natural wrapper (1C4) were acquired from the Center for Tobacco Reference Products (CTRP) at the University of Kentucky (Lexington, KY). The CORESTA monitor (CM8) was acquired from Cerulean (Milton Keynes, UK). The three HTPs IQOS, Glo, and Eclipse were acquired from Philip Morris International (PMI) (Lausanne, Switzerland), British American Tobacco (BAT) (London, UK), and R.J. Reynolds Tobacco Company (RJR) (Winston-Salem, NC), respectively.

### Instrumentation and apparatus

**Fig. 1:**
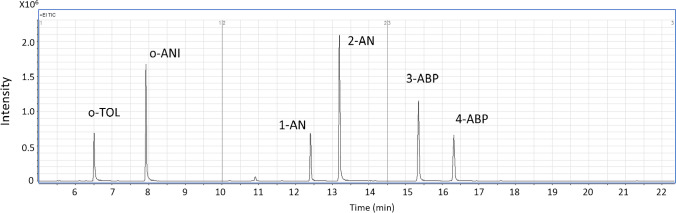
Typical chromatographic separation of the MBTFA derivatives of six aromatic amines (using a concentration of 10 ng/mL for each analyte)

The aromatic amine analyses were performed on an Agilent 7890B gas chromatograph equipped with the 7000C Triple Quad mass spectrometer system (GC/MS/MS) (Santa Clara, CA). The Agilent GC/MS/MS was coupled with the Gerstel Multipurpose sampler for SPME (Linthicum, MD). The full separation of MBTFA derivatives of six aromatic amines (Figure 1) was achieved using an Agilent DB-17 GC capillary column (30 m × 0.25 mm i.d.; 0.25 µm film thickness, Agilent Technologies) and the following gradient program: the column temperature program was started at 70 °C for 1 min and then programmed to rise to 200 °C at 10 °C/min, held for 5 min, then ramped up to 280 °C at 60 °C/min, and held for 3 min. Helium (purity > 99.9995%) at 1 mL/min flow was used as the carrier gas. The GC inlet temperature was maintained at 260 °C. All injections were made in splitless mode. The tandem mass spectrometer was operated in an electron ionization source with the multiple reaction monitoring (MRM) mode (Table 1). The temperatures of the transfer line, ion source, and quadrupoles were set at 290, 250, and 180 °C, respectively. The emission current was 35 μA.

**Table 1:** Mass spectrometric parameters for the quantification and confirmation of the MBTFA derivatives of aromatic amines in the multiple reaction monitoring (MRM) modes

	Quantitation transition (m/z)	Confirmation transition (m/z)	Collision voltage (ev)
o-TOL	203 → 134	203 → 106	15
o-ANI	219 → 150	219 → 122	10
1-AN	239 → 115	239 → 142	40
2-AN	239 → 115	239 → 142	40
3-ABP	265 → 153	265 → 168	25
4-ABP	265 → 168	265 → 141	30
o-TOL-d9	210 → 141	210 → 113	15
o-ANI-d7	226 → 157	226 → 139	10
1-AN-d7	246 → 122	246 → 149	40
2-AN-d7	246 → 122	246 → 149	40
3-ABP-d9	274 → 177	274 → 149	25
4-ABP-d9	274 → 177	274 → 149	30

### Smoke generation and collection

Before smoking, the cigarettes, cigars, and HTPs were conditioned according to the International Organization for Standardization (ISO) standard 3402:1999 [16], the CORESTA Recommended Method (CRM) 46 [17], and the CORESTA Technical Report from the CORESTA Heated Tobacco Products Task Force Group [18], respectively.

The cigarettes were smoked using a Cerulean SM450 linear smoking machine (Richmond, VA) following the standard smoking procedure ISO 3308:2012 (35 mL puff volume, 2 s puff duration, and 60 s puff frequency) [19], and the ISO Intense smoking regime (ISO 20778:2018 and ISO 20779:2018), which is 55 mL puff volume, 2 s puff duration, 30 s puff frequency, and 100% ventilation blocking [20, 21]. The cigars were smoked using a Borgwaldt LM5C linear cigar-smoking machine (Hamburg, Germany) following CRM 64, in which the puff volume is 20 mL when the cigar diameter is less than 12.0 mm or the puff volume is equal to 0.139 × d^2^ when the diameter is greater than 12.0 mm, 1.5 s puff duration, and 40 s puff frequency [22]. The HTPs were smoked using a Borgwaldt LM4E modular vaping machine following CRM 81 (55 mL puff volume, 3 s puff duration, and 30 s puff frequency) [23].

Total particulate matter (TPM) in the mainstream smoke was collected on Cambridge filter pads. Five and three cigarettes per pad were smoked under the ISO and Intense smoking regimes, respectively. One cigar per pad was smoked with CRM 64 except for 1C2 which was smoked with two cigars per pad. Three sticks of each HTPs per pad were smoked following the CRM 81 smoking regime.

### Sample preparation

The Cambridge filter pads were extracted with 10 mL 0.1 M hydrochloric acid. The mixture of aromatic amine isotopes as the internal standards was spiked into the extract solution prior to shaking for 1 h. After that, 1 mL extract solution was transferred to a 10 mL headspace vial, and then 100 µL 2.5 M sodium hydroxide and 25 µL MBTFA were added to the extract solution. The MBTFA derivatives of the aromatic amines solutions were then ready for injection into the GC/MS/MS with headspace SPME mode.

### SPME procedure

All SPME fibers were conditioned following the Supelco SPME instructions prior to their first use. The vial containing the sample extract solution was automatically transferred to the agitator and incubated at 80 °C for 2 min, at an agitator speed of 250 rpm. The 85 µm polyacrylate fiber was placed in the headspace of the sample vial to extract the MBTFA derivatives of the aromatic amines at 80 °C for 20 min before the SPME fiber was inserted into the GC injector to desorb the compounds at 260 °C for 3 min.

## Results and discussion

### SPME fiber selection

**Fig. 2:**
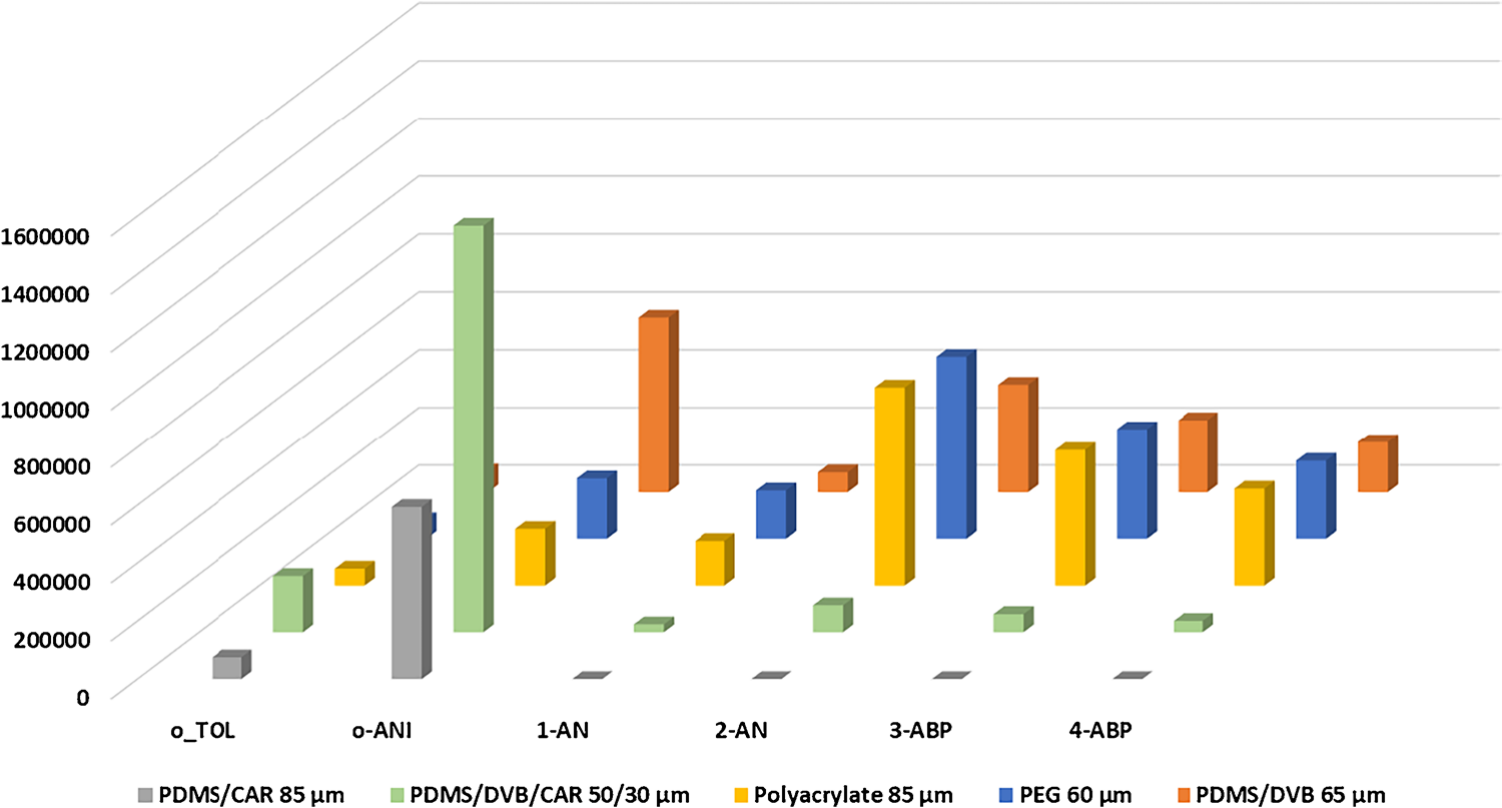
Comparison of the performances of five different SPME coating fibers for six aromatic amines

There are several commercially available SPME fibers on the market. To find the most suitable fiber for the analysis of aromatic amines, we compared the extraction efficiencies of five types of SPME fibers coated with different stationary phases (DVB/CAR/PDMS 50/30 µm, PDMS/DVB 65 µm, CAR/PDMS 85 µm, polyacrylate 85 µm, and PEG 60 µm) for the MBTFA derivatives of the aromatic amines. The spiked 7 ng/mL aromatic amines solution was used for the comparison testing with 2 min incubation time, 70 °C extraction temperature, and 20 min extraction time. The means of the responses of three replicates of each of the aromatic amines to each SPME fiber were plotted and are shown in Figure 2.

The DVB/CAR/PDMS 50/30 µm and CAR/PDMS 85 µm fibers had relatively low responses for most aromatic amines. The PDMS/DVB 65 µm and PEG 60 µm fibers had decent responses, and however, some aromatic amines had either bad peak shapes or interfering peaks. The highest extraction efficiency was achieved using the polyacrylate 85 µm fiber. Therefore, the polyacrylate 85 µm was selected for the analysis of aromatic amines in this study.

### Optimization of the condition for SPME

The extraction efficiency of headspace SPME for the aromatic amines was impacted by various experimental conditions such as solution pH, salt effect, incubation time, extraction temperature, and extraction time [24]. It was important to identify the best conditions that gave a sufficient response for aromatic amines analysis. All experiments to optimize the SPME conditions were performed in duplicate.

#### Extraction temperature profile

The fibers of headspace SPME absorb the analytes in the headspace above the samples. The volatiles and semivolatiles are present in the sample matrix, the gas phase, and the fiber coating. There are two equilibriums between the three phases: the first is between the sample matrix and the gas phase, and the second is between the gas phase and the fiber coating. The temperature influences how the analytes’ partition between the three phases [24]. A spiked 7 ng/mL aromatic amines solution was used for optimization of the extraction temperature. Extraction temperatures of 50, 60, 70, 80, and 90 °C were tested with 20 min extraction times and 2 min incubation times.

**Fig. 3:**
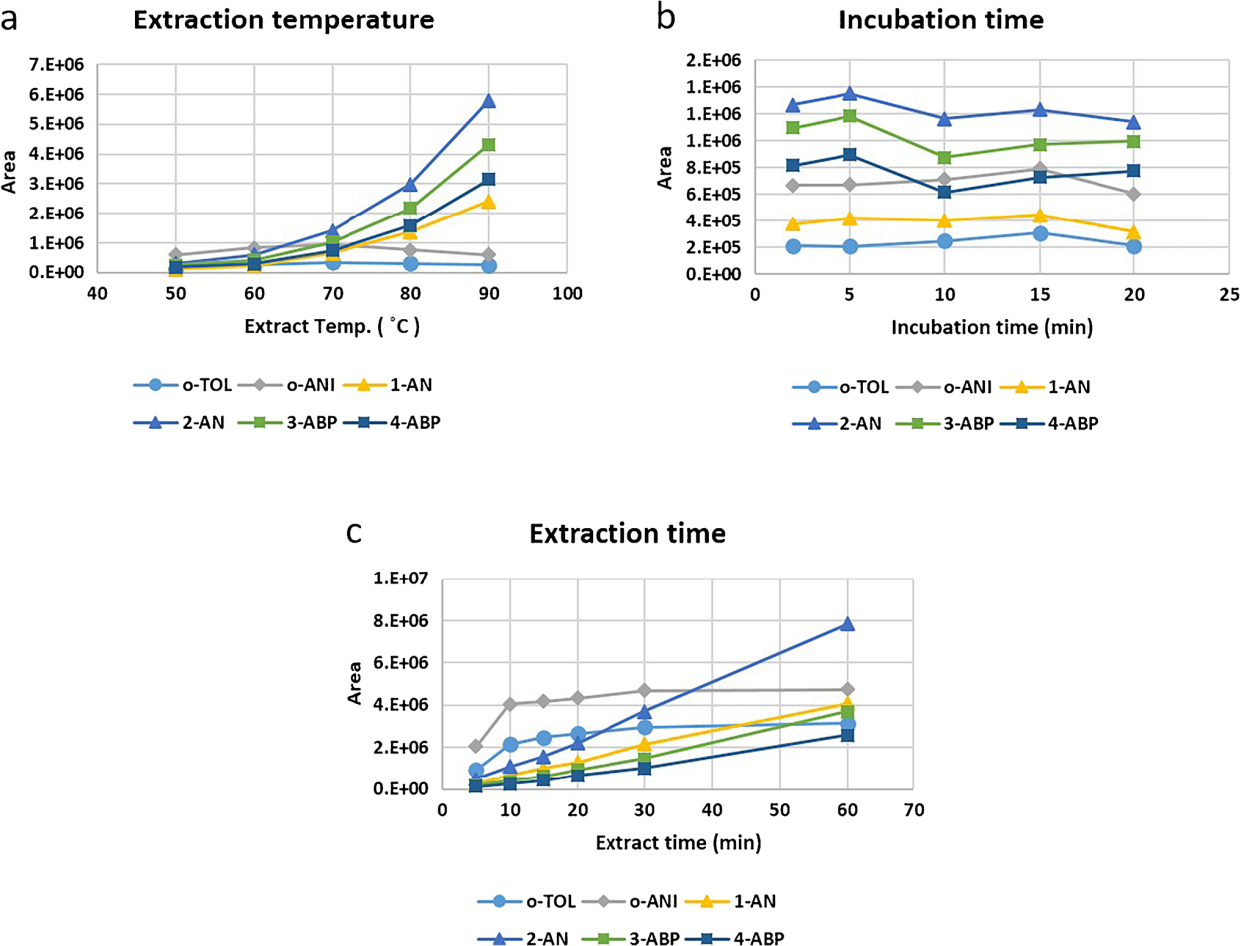
The effect of SPME conditions on peak areas of the MBTFA derivatives of aromatic amines **a** extraction temperature, **b** incubation time, and **c** extraction time

The higher temperatures could decrease the time required to reach equilibrium and increase the proportion of the analytes in the gas phase; however, an excessively high temperature might reduce the affinity of the analytes for the fiber coating. The optimum temperature for o-TOL and o-ANI is 70 °C, while the responses of 1-AN, 2-AN, 3-ABP, and 4-ABP were increased as the temperature increased (Figure 3a). To achieve a satisfactory extraction of each amine, an 80 °C extraction temperature was used in this study.

#### Effect of incubation time

The incubation time was tested at 2, 5, 10, 15, and 20 min with the 80 °C extraction temperature and 20 min extraction time. There were no significant differences between different incubation times (Figure 3b). Therefore, the 2 min incubation time was used.

#### Evaluation of extraction time

An extraction time profile was established by plotting the peak areas of each analyte against extraction time. The 5, 10, 15, 20, 30, and 60 min extraction times were tested with 80 °C extraction temperature and 2 min incubation time. The signals for all analytes increased with increasing extraction times (Figure 3c). However, it is not necessary to reach equilibrium for each analyte for quantitative analysis, as long as the fiber extract has a sufficient amount and the extraction time is the same for all analyses. Considering both the feasibility and sensitivity, a 20 min extraction time was selected.

#### Effect of extract solution pH

Adjusting the pH of the extract solution greatly improved the sensitivity of the aromatic amines analysis. The responses of the MBTFA derivatives of aromatic amines in the tobacco matrix are low under acidic conditions. Using a strong base to adjust the pH significantly increased the responses. In this study, 100 µL of 2.5 M sodium hydroxide was used to adjust the pH of the extract solutions.

### Method validation

The linearity of the method was investigated by establishing standard calibration curves of the aromatic amines. A mixture of aromatic amine isotopes was spiked in as the internal standards. The graphs of the peak area ratio versus the concentration ratio of the MBTFA derivative of each aromatic amine to its corresponding internal standard were plotted. The calibration type was linear with 1/*x* weighting, and the regression lines were not forced through the origin. All aromatic amines showed excellent linear responses (> 0.9994) (Table 2).

**Table 2:** Summary of the limits of detection (LODs), the limits of quantitation (LOQs), and calibration curve range/linearity for the six aromatic amines analyzed in this study

	o-TOL	o-ANI	1-AN	2-AN	3-ABP	4-ABP
Calibration range (ng/mL)	1.2–60.5	0.2–7.5	0.4–20.4	0.4–20.4	0.1–4.6	0.1–4.2
Linearity, *R*^2^	0.9999	0.9994	0.9999	0.9999	0.9999	0.9997
LOD (pg/mL)	96	18	23	12	20	17
LOQ (pg/mL)	320	59	78	41	67	56

The method was validated for the precision and accuracy of each analyte at different concentrations. The mainstream smoke condensate of the 2R5F reference cigarette, an ultra-low tar delivery cigarette that generates about 2 mg TPM per cigarette, was used as the matrix for the precision and accuracy test. The low and high levels of the aromatic amines with internal standards were spiked into the 2R5F smoke condensate extract solutions. A total of six replicates of each concentration level were tested on two different days. The recovery and coefficient of variation (CV%) of each analyte were 90–112% and 2.1–6.6%, respectively (Table 3).

**Table 3:** Method accuracy with the six aromatic amines spiked into 2R5F smoke condensate (*n* = 6)

	Spiked concentration	Recovery	CV
	ng/mL	%	%
o-TOL	6.05	94.9	5.4
	36.3	100.5	3.0
o-ANI	0.75	92.6	5.4
	4.49	98.9	3.0
1-AN	2.04	97.9	5.0
	12.25	102.2	2.2
2-AN	2.2	106	4.6
	12.0	112.3	2.1
3-ABP	0.46	101.1	6.6
	2.76	107.2	2.7
4-ABP	0.42	90.4	3.4
	2.49	96.9	3.2

A series of aromatic amines standards of known concentration were reacted with MBTFA and then injected into the GC/MS/MS five times to determine the limit of detection (LOD) and the limit of quantitation (LOQ). The standard deviations of the concentrations from the five injections versus the concentration of each analyte were plotted. The value of the *y*-intercept of the linear regression (*s*_0_) is the estimation of the standard deviation when the analyte is zero. The LOD and LOQ were estimated as 3*s*_0_ and 10*s*_0_, respectively [25]. The LOD and LOQ results are presented in Table 2.

### Detection of aromatic amines in the mainstream smoke of cigarettes, cigars, and HTPs

Aromatic amines are formed during the combustion of tobacco products [26]. The aromatic amines in the mainstream smoke of the reference cigarettes (1R6F, 2R5F, and CM8), reference cigars (1C1, 1C2, 1C3, and 1C4), and HTPs (IQOS, Eclipse, and Glo pro) were measured using the method developed in this study. All samples were analyzed in six replicates on two different days.

The reference cigarettes were smoked using a linear smoking machine following the standard smoking procedure ISO 3308:2012 and the ISO Intense smoking regimes. The results were consistent with the previous results from the CORESTA collaborative study (Table 4) [12, 27], except that 2R5F, which is a new reference product, was not included in the previous study. The results for 1-AN, 2-AN, and 4-ABP in the 1R6F cigarette smoke are also consistent with the certificate of analysis for 1R6F [28].

**Table 4:** The levels of six aromatic amines in the mainstream smoke of three reference cigarettes (*n* = 6)

Analytes	Results from the developed method (*n* = 6)						Results from the literature			
	2R5F		1R6F		CM8		1R6F		CM8	
Smoking regime	ISO	Intense	ISO	Intense	ISO	Intense	ISO	Intense	ISO	Intense
o-TOL (ng/cigarette)	10.4 ± 0.7	43.1 ± 6.5	41.9 ± 2.3	71.0 ± 6.6	56.7 ± 1.7	99.0 ± 9.2	36.7 ± 7.8	67.7 ± 53.4	54.1 ± 11.4	94.3 ± 75.2
o-ANI (ng/cigarette)	0.8 ± 0.1	3.0 ± 0.4	2.4 ± 0.1	4.2 ± 0.4	4.4 ± 0.1	7.5 ± 0.7	1.6 ± 0.6	2.9 ± 3.5	2.9 ± 0.7	5.3 ± 5.7
1-AN (ng/cigarette)	3.7 ± 0.2	11.7 ± 1.5	10.1 ± 0.6	16.4 ± 1.5	14.7 ± 0.6	25.3 ± 1.5	12.4 ± 2.8	21.9 ± 11.8	15.9 ± 3.4	32.5 ± 28.1
2-AN (ng/cigarette)	2.6 ± 0.2	7.0 ± 0.6	6.3 ± 0.5	9.6 ± 0.8	7.5 ± 0.3	12.1 ± 0.3	6.6 ± 1.8	12.0 ± 10.5	8.1 ± 2.1	15.9 ± 14.0
3-ABP (ng/cigarette)	0.7 ± 0.1	2.7 ± 0.4	1.7 ± 0.2	3.7 ± 0.5	1.8 ± 0.1	3.8 ± 0.4	1.6 ± 0.4	3.5 ± 3.9	1.9 ± 0.5	4.0 ± 4.7
4-ABP (ng/cigarette)	0.5 ± 0.1	1.7 ± 0.2	1.2 ± 0.1	2.2 ± 0.3	1.2 ± 0.1	2.3 ± 0.2	1.0 ± 0.3	2.4 ± 2.8	1.2 ± 0.3	2.5 ± 2.8

To compare the level of aromatic amines in cigarettes and cigars, the cigarettes and cigars were smoked following the same smoking regime, CRM 64, using a linear cigar smoke machine. The HTPs were smoked using CRM 81. The results are shown in Table 5. For all of the smoke analyses, we ran six replicates on two different days. On a per test unit basis, cigars generated more aromatic amines in the mainstream smoke than did the cigarettes.

**Table 5:** The levels of six aromatic amines in the mainstream smoke of reference cigarettes, reference cigars, and HTPs (*n* = 6)

		o-TOL	o-ANI	1-AN	2-AN	3-ABP	4-ABP
		ng/piece	ng/piece	ng/piece	ng/piece	ng/piece	ng/piece
Cigarettes	2R5F	3.8 ± 0.4	0.4 ± 0	2.0 ± 0.2	1.5 ± 0.2	0.5 ± 0.1	0.4 ± 0.1
	1R6F	34.5 ± 1.6	2.1 ± 0.1	9.7 ± 0.5	6.3 ± 0.5	1.9 ± 0.1	1.2 ± 0.1
	CM8	56.6 ± 0.5	4.5 ± 0.1	16.3 ± 0.4	8.8 ± 0.5	2.3 ± 0.1	1.4 ± 0.1
Cigars	1C1	383.2 ± 52.2	9.9 ± 1.9	97.4 ± 17.5	66.5 ± 13.2	16.3 ± 2.9	11.5 ± 2.1
	1C2	116.7 ± 5.3	4.0 ± 0.2	41.7 ± 2.8	21.5 ± 3.2	5.6 ± 0.4	3.7 ± 0.2
	1C3	334.8 ± 36.3	12.1 ± 1.2	115.7 ± 7.2	50.3 ± 6.8	17.9 ± 0.7	12.0 ± 0.7
	1C4	438.4 ± 95.4	18.7 ± 5.1	120.5 ± 9.5	48.1 ± 8.9	22.1 ± 3	14.3 ± 1.4
HTPs	Glo	< LOQ	< LOQ	< LOQ	< LOQ	< LOD	< LOD
	IQOS	< LOQ	< LOQ	< LOQ	< LOQ	< LOD	< LOD
	Eclipse	13.4 ± 2.3	0.9 ± 0.1	1.1 ± 0.2	0.6 ± 0.1	0.2 ± 0	0.2 ± 0

The contents of aromatic amines in IQOS and Glo products are below the LOQ. The Eclipse device generated a small amount of aromatic amines in the smoke. IQOS and Glo are electrically heated tobacco products (eHTPs) in which the tobacco is heated by an electrical heating device without combustion, while Eclipse is a carbon heated tobacco product (cHTP) in which the tobacco is heated by smoldering carbon to produce a nicotine-containing aerosol [18]. The combustion temperature is an important factor in the formation of aromatic amines. The content of aromatic amines was significantly reduced when the temperature was decreased [11]. HTPs heat tobacco to a temperature below 350 °C without burning the tobacco, while the burn zone temperature for traditional cigarettes and cigars is about 900 °C [29]. Therefore, the average levels of aromatic amines in HTPs were significantly reduced compared to cigarette and cigar mainstream smoke.

## Conclusions

A SPME headspace GC-MS/MS method was developed and validated for the quantitative determination of six aromatic amines in mainstream tobacco smoke. This method combines high sensitivity and selectivity with quick and simple sample preparation using a SPME coupled with triple quadrupole mass spectrometry. The new method is suitable for routine sample analyses and has been successfully applied to mainstream tobacco smoke, including cigarettes, cigars, and HTPs.
